# Liver stiffness and associated risk factors among people with a history of injecting drugs: a prospective cohort study

**DOI:** 10.1186/s13011-024-00603-z

**Published:** 2024-03-26

**Authors:** Karl Trygve Druckrey-Fiskaaen, Jørn Henrik Vold, Tesfaye Madebo, Håvard Midgard, Olav Dalgard, Rafael Alexander Leiva, Lars T. Fadnes, for the INTRO-HCV Study Group, Vibeke Bråthen Buljovcic, Jan Tore Daltveit, Trude Fondenes, Per Gundersen, Beate Haga Trettenes, Siv-Elin Leirvåg Carlsen, Mette Hegland Nordbotn, Maria Olsvold, Marianne Cook Pierron, Christine Sundal, Maren Borsheim Bergsaker, Eivin Dahl, Tone Lise Eielsen, Torhild Fiskå, Marianne Larssen, Torgeir Gilje Lid, Eirik Holder, Ewa Joanna Wilk, Mari Thoresen Soot

**Affiliations:** 1 Bergen Addiction Research, Department of Addiction Medicine, Haukeland University Hospital, Bergen, Norway; 2 Department of Global Public Health and Primary Care, University of Bergen, Bergen, Norway; 3 Division of Psychiatry, Haukeland University Hospital, Bergen, Norway; 4 Department of Respiratory Medicine, Stavanger University Hospital, Stavanger, Norway; 5 Department of Clinical Science, University of Bergen, Bergen, Norway; 6 Department of Gastroenterology, Oslo University Hospital, Oslo, Norway; 7 Department of Infectious Diseases, Akershus University Hospital, Lørenskog, Norway; 8 Department of Infectious Diseases, Haukeland University Hospital, Bergen, Norway

**Keywords:** Chronic liver disease, Opioid agonist treatment, Substance use disorder, Injecting drug use, Liver stiffness measurement, Elastography, Hepatitis C, Steatotic liver disease, Prospective cohort

## Abstract

**Background:**

Persons with opioid use disorders (OUD) and persons with substance use disorders (SUD) who inject substances have a reduced life expectancy of up to 25 years compared with the general population. Chronic liver diseases are a substantial cause of this. Screening strategies based on liver stiffness measurements (LSM) may facilitate early detection, timely intervention, and treatment of liver disease. This study aims to investigate the extent of chronic liver disease measured with transient elastography and the association between LSM and various risk factors, including substance use patterns, hepatitis C virus (HCV) infection, alcohol use, body mass index, age, type 2 diabetes mellitus, and high-density lipoprotein (HDL) cholesterol among people with OUD or with SUD who inject substances.

**Methods:**

Data was collected from May 2017 to March 2022 in a cohort of 676 persons from Western Norway. The cohort was recruited from two populations: Persons receiving opioid agonist therapy (OAT) (81% of the sample) or persons with SUD injecting substances but not receiving OAT. All participants were assessed at least once with transient elastography. A linear mixed model was performed to assess the impact of risk factors such as HCV infection, alcohol use, lifestyle-associated factors, and substance use on liver stiffness at baseline and over time. Baseline was defined as the time of the first liver stiffness measurement. The results are presented as coefficients (in kilopascal (kPa)) with 95% confidence intervals (CI).

**Results:**

At baseline, 12% (*n* = 83) of the study sample had LSM suggestive of advanced chronic liver disease (LSM ≥ 10 kPa). Advanced age (1.0 kPa per 10 years increments, 95% CI: 0.68;1.3), at least weekly alcohol use (1.3, 0.47;2.1), HCV infection (1.2, 0.55;1.9), low HDL cholesterol level (1.4, 0.64;2.2), and higher body mass index (0.25 per increasing unit, 0.17;0.32) were all significantly associated with higher LSM at baseline. Compared with persistent chronic HCV infection, a resolved HCV infection predicted a yearly reduction of LSM (-0.73, -1.3;-0.21) from baseline to the following liver stiffness measurement.

**Conclusions:**

More than one-tenth of the participants in this study had LSM suggestive of advanced chronic liver disease. It underscores the need for addressing HCV infection and reducing lifestyle-related liver risk factors, such as metabolic health factors and alcohol consumption, to prevent the advancement of liver fibrosis or cirrhosis in this particular population.

**Supplementary Information:**

The online version contains supplementary material available at 10.1186/s13011-024-00603-z.

## Introduction

Persons with substance use disorders (SUD) such as opioid use disorder (OUD) and persons who inject substances have a substantially shorter life expectancy than the general population [1–3]. This is attributable to a high burden of diseases, including infectious diseases, chronic liver disease, cancer, cardiovascular diseases, and substance-related deaths [4–7]. For persons with OUD, opioid agonist therapy (OAT) is an essential treatment approach, which almost halves mortality in this population, yet the mortality remains high within this group [8, 9]. Diseases of the digestive system, such as chronic liver disease, significantly contribute to the morbidity and mortality of persons with OUD or injecting substance use [10, 11]. In a systematic review, the standard mortality rate related to diseases of the digestive system was estimated to be 3.4 for males and 7.9 for females [10]. In an autopsy study from Norway of 122 patients who died during OAT treatment, at least one liver disease was identified in 84% of the decedents [11]. Amidst a growing global prevalence of liver cirrhosis, there is a need to improve the understanding of the risk factors for liver disease and their progression among persons with OUD or injecting substance use [12, 13] to achieve timely detection and prevent disease progression [14].

Viral hepatitis, high alcohol consumption, and metabolic dysfunction such as obesity, low high-density lipoprotein (HDL) cholesterol and type 2 diabetes mellitus are acknowledged as frequent causes of chronic liver disease worldwide [15–17]. Among persons with SUD, polysubstance use, including alcohol [8, 18] and intravenous substance use, have been associated with liver diseases [19]. Polysubstance use is common among persons with SUD and predicts more severe comorbidities such as suicidal attempts, liver diseases or overdose deaths than among those with mono-substance use [20–22]. Multiple risk factors for chronic liver disease significantly increase the likelihood of compensated advanced chronic liver disease (cALCD), leading to liver cirrhosis and hepatocellular carcinoma [23, 24]. However, there is less knowledge on the prevalence and magnitude of the association of various risk factors with the risk of chronic liver disease among persons with SUD.

Persons with SUD have a higher barrier to seeking primary healthcare compared to the general population, leading to delayed initiation of diagnostics and treatments in a primary care setting [25]. To ensure early detection and prevent liver disease from progressing among persons at risk, case-finding procedures and risk assessment strategies may help identify persons needing assessment for treatment and follow-up [14]. Non-invasive methods such as liver elastography have largely replaced liver biopsy for staging liver diseases such as liver fibrosis or cirrhosis [26, 27]. Liver stiffness measurements (LSM) can identify persons at risk of developing cALCD, and repeated measurements have a prognostic value for determining the risk of disease progression [16, 28]. LSM correlates with the histological stage of liver disease, portal hypertension, and the risk of liver decompensation events and death [26, 29, 30]. Thus, elastography is an essential tool to identify patients at risk and to determine the stage of liver disease and prognosis.

This study aims to investigate the prevalence of cALCD as assessed by transient elastography and to estimate how potential risk factors such as HCV infection, substance use patterns, and metabolic risk factors impact the LSM among persons receiving OAT or persons with SUD injecting substances but not receiving OAT. We hypothesise that metabolic risk factors and polysubstance use, in addition to HCV infection and alcohol use, will predict increased LSM. We further hypothesise that the participants in our study face several simultaneous risk factors for increased LSM.

## Material & method

### Study setting and sample

**Fig. 1:**
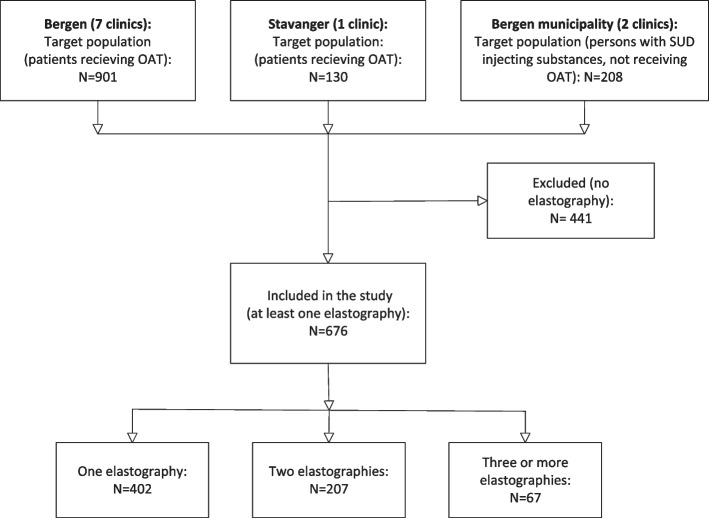
Overview of the recruitment and inclusion of participants in the study. The figure indicates which treatment facilities the participants were recuited from and the number of persons in the target population. The figure further indicates the number of participants with one or more elastographies

We used data from a prospective cohort nested in the INTRO-HCV study in Bergen and Stavanger, Norway [31]. Data were collected from May 2017 to March 2022 as a part of annual health assessments, and participants were recruited from eight OAT outpatient clinics in Bergen and Stavanger and two municipal outpatient SUD treatment centres in the Bergen Municipality. The cohort has also been described previously [32]. The target populations were persons receiving OAT or persons with SUD injecting substances but not receiving OAT. Participants recruited from the OAT clinics were all diagnosed with opioid dependency (F10.2) according to World Health Organization International Classification of Diseases version 10 [33] and received OAT at OAT clinics in Bergen or Stavanger. Participants recruited from the municipal SUD treatment centres had SUDs and injected substances but did not receive OAT. Persons receiving OAT accounted for 81% of the study sample. Supplementary Table 1, Additional File 1 shows the baseline characteristics for the participants recruited from OAT clinics and municipal SUD clinics separately. Persons were recruited by the staff at the participating clinics; see Fig. 1 for an overview of recruitment and inclusion. Persons in the target population were offered an initial health assessment, informed about the study, and asked to consent to participation. Participants were eligible for our study if they had completed at least one elastography measurement at a participating OAT clinic or municipal SUD treatment centre, one annual health assessment and consented to participate. We included 676 persons who completed at least one annual health assessment. All participants completed at least one liver stiffness measurement, and 274 persons had completed two or more measurements, rendering 345 repeated liver stiffness measurements (Fig. 1). The median time interval between the first and last liver stiffness measurements was 14 months (interquartile range (IQR): 11–19).

### Data collections

All participants included in the study underwent an annual health assessment, encompassing the gathering of blood samples, liver elastography, assessment of substance use for the past twelve months, and sociodemographic and clinical data collection. Blood samples were systematically annually screened for hepatitis B and C viruses as well as human immunodeficiency virus (HIV). In addition, a thrombocyte count and analysis of liver transaminases and cholesterol including high- and low-density lipoproteins, glycated haemoglobin, and creatinine were performed. We analysed for triglycerides if the participants were fasting. Educational attainment and other demographic information were collected at the first health assessment. The data were collected using electronic data collection software (CheckWare, Checkware AS, Norway) under the supervision of research nurses and stored in a health register. Baseline was defined as the time of the first liver stiffness measurement. Blood samples were analysed at the Department of Laboratory Medicine, Haukeland University Hospital, Bergen, Norway, and the Department of Medical Biochemistry and Microbiology, Stavanger University Hospital, Stavanger, Norway (both accredited by ISO standard 15189).

#### Measuring liver stiffness

Trained research nurses performed transient elastography according to the European Association for the Study of the Liver Guidelines [34] using a Fibroscan® mini 430 with a medium probe to measure the participants’ liver stiffness. Before the examination, the participant had been instructed to fast for at least 2 h. The liver stiffness estimate is reported in kPa as a median score of at least ten elastography measurements with an interquartile range/ median of < 30%. LSM values < 10 kPa in the absence of other known clinical/imaging signs rule out cACLD; values between 10 and 15 kPa are suggestive of cACLD; values > 15 kPa are highly suggestive of cACLD [27].

### Definition of study variables

The study sample was categorized into the following age groups: below 30 years of age, 30–39 years, 40–49 years, 50–59 years, and 60 years and older. Body mass index (BMI) was defined as body weight in kilograms divided by height in meters squared. The housing situation in the 30 days prior to the assessment was defined as “stable” if living in an owned or rented home or being incarcerated. An “unstable” housing situation was defined as living in a homeless shelter with family or friends or on the street. The OAT medication (if any) was defined as the type of medication used at baseline.

We estimated the self-reported substance use during the past 12 months prior to the health assessment using a Likert scale for each substance class, including alcohol, non-prescribed benzodiazepines, tobacco, non-prescribed opioids, cannabis, and stimulants including amphetamines or cocaine. The scale ranges from zero to five points, where zero represents never using, one represents less than monthly, two represents one to three days per month, three represents one to three days per week, four represents more than three days per week, and five represents daily use of a substance. Regular use was defined as weekly use (≥ 3 points). To investigate the impact of the intensity of polysubstance use on LSM, we built an illegal substance use severity index (iSUSI) based on the sum score responses for non-prescribed benzodiazepines, cannabis, illicit opioids, and stimulants (cocaine and amphetamines). The sum score was divided by 20 to generate a continuous range from 0 to 1, where zero indicates no use and one indicates daily use of all substance classes. The data collection software only allowed valid responses to each substance and prompted empty questions before submission to minimize missing data.

We systematically screened participants for chronic infectious blood-borne virus diseases in the blood samples including HCV (using HCV ribonucleic acid by polymerase chain reaction), hepatitis B virus infection (hepatitis B virus surface antigens), and HIV (HIV antigen/antibodies). We defined that an HCV infection was cured if HCV RNA was negative once following successful HCV treatment or by spontaneous HCV clearance.

Based on the available data from the cohort we included the following cardio-metabolic risk factors in the analysis [17]: Blood sugar levels were defined as “elevated” if glycated haemoglobin (HbA1c) was ≥ 48 mmol/mol [35]. HDL cholesterol < 1.3 mmol/L for women and < 1.0 mmol/L for men were defined as “low” [36, 37]. Obesity was defined as BMI ≥ 30 kg/m2 [38].

### Statistical analysis

We used Stata/SE17 (StataCorp, TX, USA) and IBM SPSS version 26 (International Business Machines, Chicago, USA) for descriptive statistics including means and standard deviation (SD), and for linear mixed model analyses. The threshold for statistical significance was set to *p* < 0.05 unless otherwise stated.

Any missing values in the exposure variables, including HCV RNA, BMI, alcohol, benzodiazepine, cannabis, tobacco, opioid and stimulant use, HbA1c, injecting behaviour, and HDL cholesterol, were considered “missing at random” when performing the expectation–maximization algorithm. Missing values were identified in 8.0% of the exposure variables, and all were replaced with estimated values using the expectation–maximization algorithm [39]. A total of 17% of the HCV RNA results and 14% of the HDL cholesterol results were replaced with estimated values. The distribution of missing values among participants are shown in Supplementary Table 2, Additional File 1.

We performed a linear mixed model analysis to assess the association of age, alcohol consumption, use of illegal substances, BMI, HCV, low HDL cholesterol, and elevated HbA1c (exposure variables) on liver stiffness measurements (continuous outcome variable) at baseline and to what extent they were associated with changes in liver stiffness over time. This is presented with 95% confidence interval (CI). We did not adjust HDL cholesterol or HbA1c values for the use of lipid-lowering drugs or antidiabetic drugs, as this information was not provided by the participants. Except for HCV infection status, due to relatively stable exposure variable values over time [40], all these variables including age group were kept constant at the baseline level in predicting the level and changes in the outcome variable. Interactions between these variables and time were added to the model to investigate whether exposure variables predicted changes in outcome. All available liver stiffness measurements were included. The model used a random intercept and fixed slope with the estimator set to restricted maximum likelihood. Time was defined as years from baseline.

Using the exposure variables as in the linear mixed model, we performed a sensitivity analysis with a dichotomous outcome variable of liver stiffness based on the threshold of 10 kPa (Supplementary Table 3, Additional File 1). The threshold of ≥ 10 kPa indicates that patients with liver stiffness measurements equal to or above this cut-off have an increased risk of liver disease, according to Baveno VII criteria [27]. Liver stiffness measurements using a medium probe in persons with obesity may produce unreliable results overestimating the liver stiffness [34, 41]. Thus, we ran a sensitivity analysis which only included persons with BMI < 30 kg/m^2^ (Supplementary Table 4, Additional File 1). We further performed sensitivity analyses by adding sex as an exposure variable (Supplementary Table 5, Additional File 1).

## Results

### Participants’ characteristics at baseline

At baseline, a total of 185 (27%) of the participants were females, and the mean age was 43 years (SD: 11 years); 542 patients (81%) received OAT (Table 1). Most participants smoked tobacco at least once a week (*n* = 587, 93%), and 163 (26%) reported drinking alcohol one or more days a week. The mean of the iSUSI was 0.36 (SD 0.23). A total of 344 persons (55%) had injected substances during the twelve months leading up to baseline, 310 participants (46%) were HCV RNA positive, less than five (0.7%) were infected with hepatitis B virus, and less than five (0.7%) had a HIV infection (all persons with hepatitis B virus and HIV were co-infected with HCV).

**Table 1:** Baseline characteristics of the participants (*n* = 676)

*Sex n (%)*	
Females	185 (27)
Males	491 (73)
*Age groups, years n (%)*	
18—29	81 (12)
30—39	200 (26)
40 – 49	200 (26)
50—59	160 (24)
60 +	35 (5)
Age, mean (SD)	43 (11)
BMI kg/m^2^, mean (SD)	25 (4.8)
*Housing situation past 30 days*^*1*^* n (%)*	
Stable	587 (87)
Unstable	89 (13)
*Highest completed education n* (%)	
Not finished basic education^2^	34 (5)
Finished basic education^2^	310 (46)
High school^3^	266 (39)
< 3 years of higher education	51 (8)
> 3 years of higher education	15 (2)
*Current OAT medication n (%)*	
Methadone	220 (33)
Buprenorphine-based	322 (48)
Naltrexone	1 (0.2)
None	133 (20)
*Regular substance use*^*4*^* n (%)*	
Alcohol	163 (26)
Tobacco	587 (93)
Cannabis	324 (51)
Stimulants	187 (30)
Opioids	99 (16)
Benzodiazepines	236 (37)
iSUSI^5^, mean (SD)	0.36 (0.23)
Injected past 12 months^6^ n (%)	344 (55)
Hepatitis B antigen positive n (%)	< 5 (0.7)
HIV positive n (%)	< 5 (0.7)
*Hepatitis C status n (%)*	
Antibody negative	56 (8)
Antibody positive, RNA negative	251(37)
RNA positive	310(46)
Missing information^7^	59 (9)
*Blood tests mean (SD)*	
Alanine transaminase U/L	53 (67)
Aspartate transaminase U/L	49 (49)
Thrombocytes 10^9^/L	246 (80)
Estimated glomerular filtration rate ml/min/1,73m^2^	107(26)
*Liver disease risk factors and markers* n (%)	
HbA1c elevated^8^	14 (2)
Low HDL^9^	173 (26)
Low thrombocytes^10^	62 (9)
Obesity^11^ ≥ 30 kg/m^2^	104 (15)
Liver stiffness^12^ ≥ 10 kPa	83 (12)

Missing values are not included in the percentages

^1^Living in an owned or rented home or being incarcerated was defined as a stable housing situation, while living in a homeless shelter, with family or friends, or on the street was defined as an unstable housing situation

^2^In Norway, the first ten school years are mandatory for all pupils

^3^Grades 11–13

^4^Substance used more regularly than once a week for the past 12 months

^5^The illegal substance use severity index is a continuous variable ranging from 0–1, were (1) indicates the effect of maximum substance use and (0) indicates the effect of no substance use

^6^Intravenous injection of drugs at least once during the past 12 months

^7^No information on hepatitis C status registered

^8^Defined as HbA1c > 48 mmol/mol, which is diagnostic of diabetes [35]

^9^Values below 1.3 mmol/L for women and 1.0 mmol/L for men are defined as low [36, 37]

^10^Thrombocyte count of < 150 × 10^9^/L is defined as low and is associated with an increased risk of portal hypertension [27]

^11^BMI ≥ 30 kg/m^2^ increases the risk of severe liver disease outcome [38]

^12^Liver stiffness ≥ 10 kPa indicates an increased risk of compensated advanced chronic liver disease [27]

### Liver stiffness measurements at baseline and over time

**Fig. 2:**
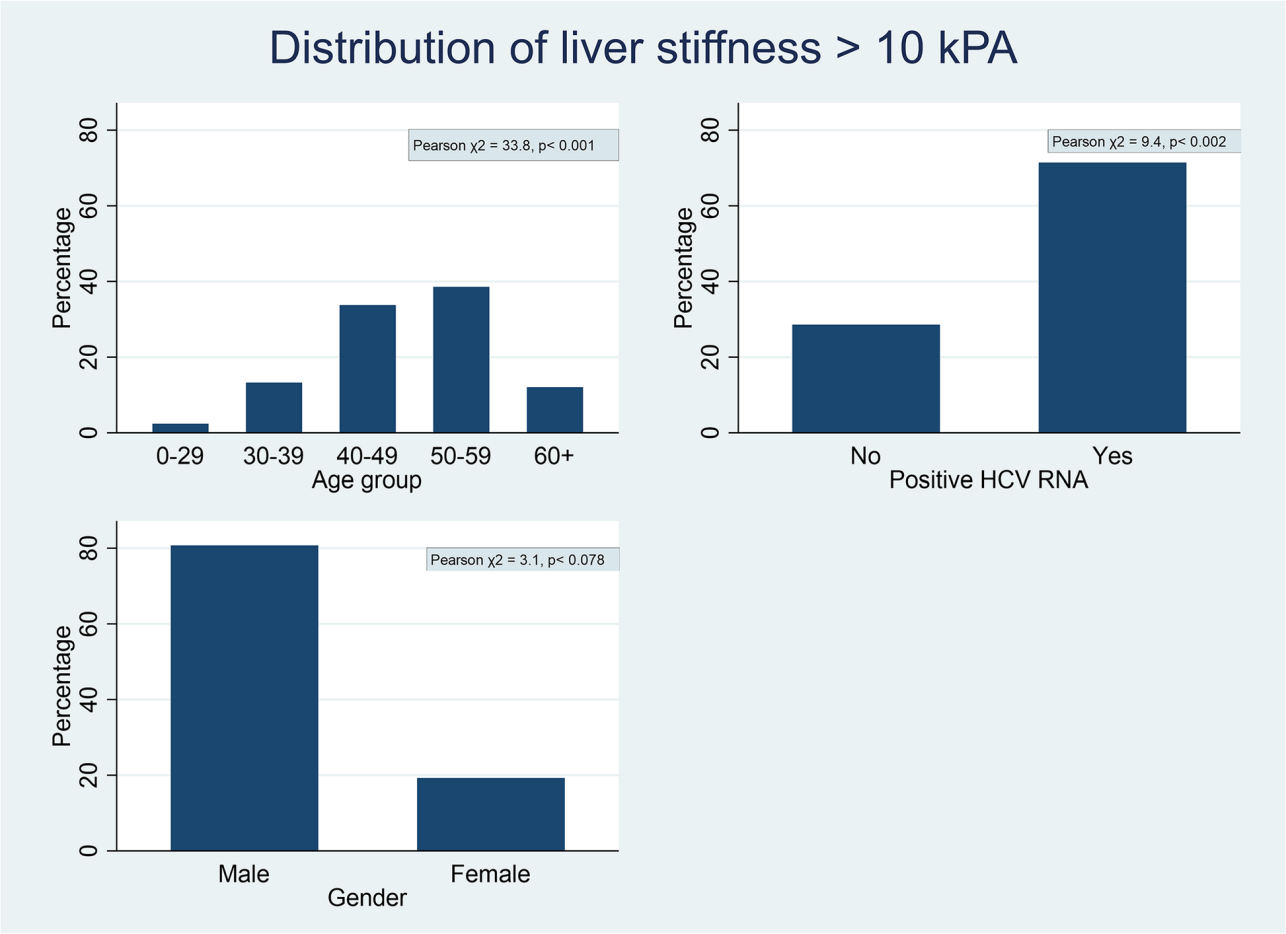
Distribution of liver stiffness according to age, HCV status, and sex. The figure indicates how the increased risk of liver disease, indicated by liver stiffness ≥ 10 kPa, is distributed between age groups, HCV infection status, and sex. The Pearson χ^2^ test indicates that there were significant differences between age groups (χ^2^ = 33.8, *p* < 0.001) and by HCV infection status (χ^2^ = 9.4, *p* < 0.002), but not between females and males (χ^2^ = 3.1, *p* < 0.078)

**Fig. 3:**
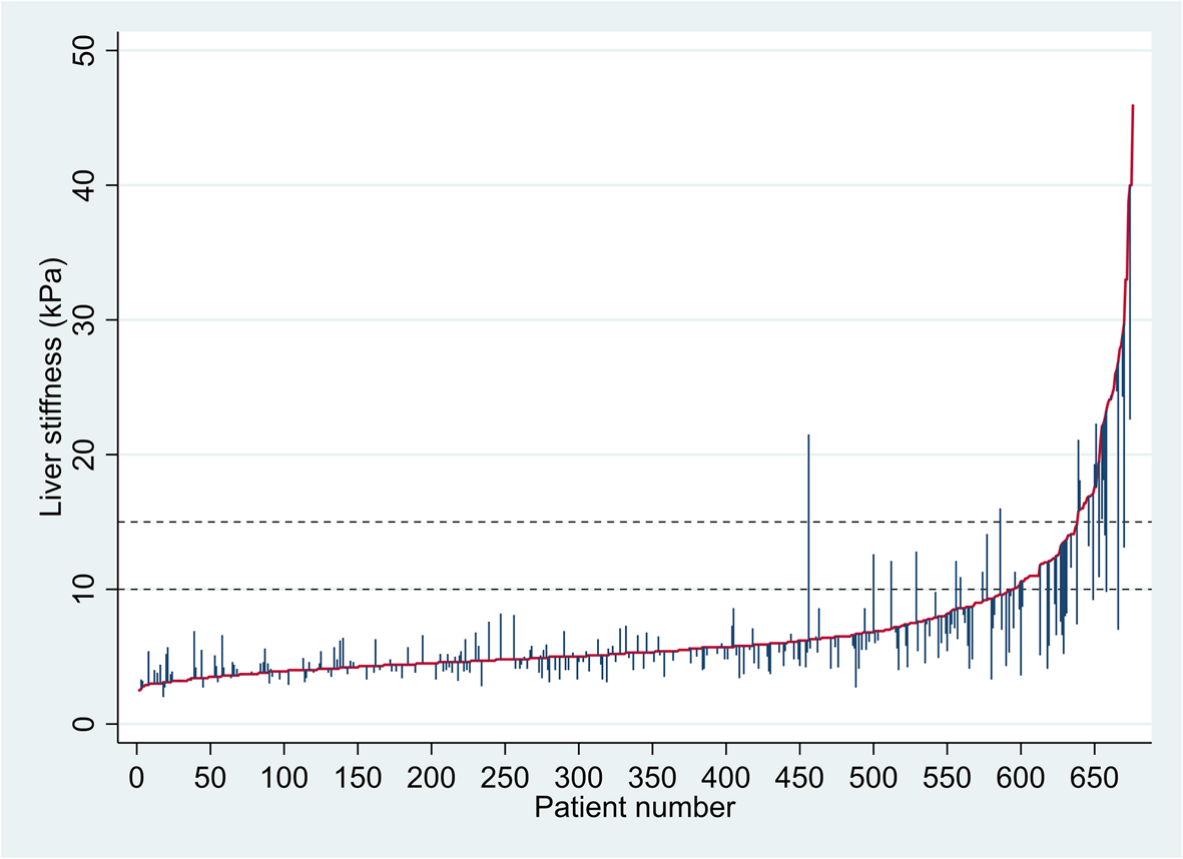
Distribution of liver stiffness measurements and its changes among the 676 patients included in the study. The red line indicates the baseline measurements sorted in incremental order. Spikes away from the red line indicate the change in liver stiffness from the first to the last measurement measured with elastography (in kPa). The black dashed line at 10.0 kPa indicates liver stiffness suggestive of compensated advanced chronic liver disease, whereas the black dashed line at 15.0 kPa indicates the threshold for liver stiffness highly suggestive of compensated advanced chronic liver disease such as liver fibrosis or cirrhosis [27]

The mean liver stiffness at baseline was 6.8 kPa (SD 5.1), and 12% had liver stiffness values ≥ 10 kPa. Figure 2 shows the distribution of liver stiffness (≥ 10 kPa) according to age groups, HCV status, and sex. For the 274 persons who completed more than one liver stiffness measurement, the mean liver stiffness at the time of the last measurement was 6.2 kPa (SD 3.8), and 9% (*n* = 25) of participants had liver stiffness measurement ≥ 10 kPa (Fig. 3).

### Liver stiffness and associated factors at baseline and over time

The liver stiffness was 1.0 kPa (CI 0.68; 1.3) higher for each ten-year increase of age at baseline (Table 2). At baseline, having a chronic HCV infection was associated with a 1.2 kPa (CI 0.55;1.9) higher liver stiffness level compared to persons with negative HCV PCR, whereas lower levels of HDL cholesterol and higher BMI were associated with a 1.4 kPa (CI 0.64;2.2) and an 0.25 (CI 0.17;0.32) higher level of liver stiffness, respectively. Regular use compared to no or non-regular use of alcohol was associated with a 1.3 kPa (CI 0.47;2.1) higher liver stiffness. The overall time trend for LSM among persons in the sample, with two or more measurements, was an increase of 2.8 kPa (CI 0.57; 5.1) per year. Achieving sustained virological response of HCV infection was associated with a decreasing liver stiffness over time by -0.73 kPa per year (CI -1.3; -0.21).

**Table 2:** Adjusted linear mixed model of liver stiffness (measured in kPa, *n* = 676 )

Fixed effects	Effect estimate baseline	Time trend (per year)
	Estimate (95% CI)	Estimate (95% CI)
Yearly LSM change	-	2.81 (0.56; 5.0)*
Age per 10 years increase	1.0 (0.68; 1.3)*	-0.18 (-0.45; 0.088)
Regular alcohol use^a^	1.3 (0.46; 2.0)*	-0.046 (-0.67; 0.58)
High substance use^b^	0.66 (-0.96; 2.3)	-0.48 (-1.9; 0.94)
Body mass index (kg/m^2^)	0.25 (0.17; 0.32)*	-0.079 (-0.14; -0.013)*
Hepatitis C RNA positive^c^	1.2 (0.54; 1.9)*	-
Low HDL Cholesterol^d^	1.4 (0.64; 2.2)*	-0.55 (-1.2; 0.08)
Elevated HbA1c^e^	3.1 (0.68; 5.5)*	4.6 (2.3; 6.9)*
Hepatitis C status change^f^	-	-0.73 (-1.3; -0.21)*

The constant term (β_0_) was -5.4 (CI -8.0; -2.8). Except for HCV status, the time trend indicates the effect of the variable remaining at baseline levels over time. 274 participants had two or more liver stiffness measurements. Significantly associated (*p* < 0.05) variables are labelled with bold text and an asterisk

^a^Using alcohol on one or more days per week during the past 12 months

^b^The iSUSI is a continuous variable ranging from 0–1, were (1) indicates the effect of maximum substance use and (0) indicates the effect of no substance use

^c^Hepatitis C virus RNA positive at baseline

^d^Below 1.3 mmol/L for women and 1.0 mmol/L for men

^e^Above 48 mmol/mol, indicating type 2 diabetes

^f^Resolved hepatitis C infection compared with no change in HCV infection status from baseline to the following liver stiffness measurements

Comparable results were seen in the sensitivity analysis with a dichotomised outcome variable (Supplementary Table 3, Additional File 1). The sensitivity analysis of the mixed model, only including participants with BMI < 30 kg/m^2^ indicated comparable results except for regular alcohol use, which was not associated with LSM (Supplementary Table 4, Additional File 1). There were no significant effects of adding sex as an exposure variable in the linear mixed model (Supplementary Table 5, Additional File 1). No substantial changes in the results were seen when all persons with a missing variable were excluded from the analysis (Supplementary Tables 6 and 7, Additional File 1). Regular tobacco use (at least once a week) did not significantly predict changes in LSM (Supplementary Table 8, Additional File 1).

## Discussion

Among the 676 persons receiving OAT or reporting injection of substances in this study, 12% had a likely cALCD. The most prevalent risk factors for chronic liver disease were HCV infection (46%), low HDL cholesterol (26%), alcohol consumption at least once a week (26%) and obesity (15%). At baseline, HCV infection, regular alcohol use, higher age, low HDL cholesterol levels, and higher BMI were associated with higher liver stiffness. The time trend analyses showed that sustained virologic response from HCV infection was associated with a significant decrease in liver stiffness. In contrast persistently elevated HbA1c levels were associated with a significant increase in liver stiffness.

In our sample, almost half the participants had a current HCV infection (HCV RNA positive) at baseline. In total, 83% were HCV antibody positive. In a global review from 2017, about one-half of persons who inject substances in Western Europe were HCV antibody positive [42]. In contrast, another study from 2018 found HCV-antibody positive rates of up to 90 percent in similar populations in high-income countries [10]. The treatment of HCV has received considerable international attention, particularly after the introduction of direct-acting antiviral medication [43]. As indicated by our study, HCV infection was associated with increased liver stiffness whereas treatment for HCV infection was associated with decreased liver stiffness. The magnitude of the associations was in line with observations in other studies [44]. However, there are few longitudinal studies on the impact of HCV treatment on LSM among persons with SUD who have a history of injecting substances [45]. Our study provides additional knowledge on the impact of HCV treatment on the risk of liver disease among persons with SUD. Integrated models of care have been shown to increase the uptake of HCV treatment among persons with SUD and injective behaviour [46]. Flexible, tailored and culturally informed interventions targeted at specific populations with HCV may facilitate HCV screening and treatment [47].

Our study indicates that elevated BMI and low HDL are relevant risk factors for increased LSM: One in six participants were obese, one in four had low HDL and higher BMI and low HDL were significantly associated with higher LSM at baseline. This indicates a need for lifestyle interventions to reduce metabolic risk. A systematic review of physical activity among persons receiving OAT concluded that regular physical activity improved physical fitness, mental health, and substance use [48]. Robust studies on the efficacy of dietary interventions for persons with SUD are lacking, but a review of five studies indicates that interventions may improve dietary outcomes [49]. Most included studies reported low adherence, often due to health-related issues, homelessness, lack of transportation and lack of follow-up [48, 49]. The frequency of obesity is lower in our sample compared to a cohort recruited from the Norwegian general population in which one-quarter of the participants were obese [50]. Compared to the findings of a systematic review on the prevalence of metabolic syndrome among persons with alcohol use disorder, which estimated a 38% prevalence of obesity and an 8% prevalence of low HDL, our sample had fewer participants with obesity. However, the prevalence of low HDL was higher [51]. In comparison, in a sample of 122 patients on methadone maintenance therapy recruited from an outpatient clinic in Barcelona, Spain, the prevalence of low HDL was 52%, and obesity was 27% [52]. A possible explanation for the lower prevalence of obesity in our population is the weight-lowering effect of stimulants among the one-third of the participants who reported regular use of stimulants [53]. Methadone as an agonist treatment to recover from opioid use disorder has been associated with mild to moderate weight gain [54]. As one-third of our sample received methadone the weight-gaining effect of methadone in our sample probably is lower than in a sample of persons solely receiving methadone.

The frequency of alcohol consumption within the study sample was not particularly high; 26% reported using alcohol at least once a week. In comparison, in the yearly report on all patients receiving OAT in Norway, 9,1% of the sample reported drinking amounts of alcohol during the last four weeks that resulted in a feeling of intoxication [55].

Low heterogeneity impeded the analysis of the effects of HbA1c and tobacco use on LSM. In our study sample, two percent had elevated HbA1c levels, and 93% smoked tobacco at least once a week. Tobacco use was not included in the main analysis to avoid introducing and adjusting for several variables with low heterogeneity, increasing random variation and the risk of residual confounding. A supplementary analysis indicated a non-significant association of at least weekly tobacco use with lower LSM. This is contradicted by findings from the general population, indicating growing evidence that tobacco smoking is associated with the development and progression of liver disease [56]. Among persons aged 55 and younger with alcohol-related cirrhosis, smokers had a 5-year survival rate of 42% compared to a 73% survival rate in non-smokers [57]. An estimated 85% of persons with SUD are smoking tobacco [58] Thus, tobacco smoking may be an important risk factor for liver disease in the SUD population that warrants further investigation.

The existence of multiple risk factors for liver disease in this cohort complicates causal inferences. In addition to injection-related diseases such as HCV infection, participants often used alcohol, smoked tobacco, and were obese. Nonetheless, the results of this study highlight the importance of continuous monitoring and regular assessments to identify possible liver disease development and progression [14, 59]. Integration of HCV treatment at OAT clinics and community care clinics has demonstrated an increased uptake of HCV treatment [46]. Integrating lifestyle interventions at OAT clinics and community care clinics could possibly improve adherence to and feasibility of such interventions.

The Baveno criteria define LSM cut-offs of 10 and 15 kPa and the rule-of-five to determine the risk of liver disease [13, 27]. From a clinical point of view, it may make the most sense to see this from a categorical perspective and assess LSM in relation to a certain cut-off; it has been demonstrated that persons with liver stiffness > 9.5 kPa have a significantly lower five-year survival rate [60]. However, this requires a longer observation period. On the other hand, one could assume that such differences would progress over time and that shorter observation time could provide estimates on the trend of LSM development, and that LSM later could progress past different cut-offs. A continuous analysis of factors affecting LSM could capture future relevant differences.

The strength of our study is the inclusion of all persons willing to participate at opioid agonist therapy clinics and municipal low-threshold centres. Persons with SUD are considered a hard-to-reach population with standard care and are often excluded from liver stiffness studies. However, integrated care and treatment have been shown to counteract low participation within this group [46]. The population of our study is comparable to the Norwegian population of persons who receive OAT, with about one-third females. However the mean age of our population was 43 years versus 47 years among persons receiving OAT [61]. A limitation is that we had two or more measurements for less than half of the population, limiting the possibility of concluding from the longitudinal results on change over time. Transient electrography was only performed using the medium probe. Liver stiffness measurements using a medium probe in persons with obesity may produce unreliable results overestimating the liver stiffness [34, 41]. The use of illegal substances was reflected in the aggregated iSUSI index. By using this index as a predictor in the regression analysis, we could not determine the associations between LSM and specific substances. Since there was no observed association across all substances combined and considering that substances were not measured using biological markers, we did not assess the associations with liver stiffness measurement (LSM) for individual substances except alcohol. As tobacco smoking was nearly universal within this cohort with a low degree of heterogeneity, assessing the association to liver stiffness was unfortunately not possible. Our model indicated that HbA1c was a strong predictor of higher liver stiffness. The generalizability of this finding is limited as the result is based on fourteen persons in our sample. Due to factors inherent in the way of life of persons with SUDs, measurements and tests were not all performed on the same day and at the same time intervals.

## Conclusions

Our study showed that 12% of the study sample had liver stiffness values indicating an increased likelihood of chronic liver disease at baseline. Achieving sustained virologic response from HCV was associated with decreased liver stiffness over time. Higher liver stiffness at baseline was observed among people with low levels of HDL cholesterol, higher age, regular alcohol use and being overweight. These results highlight the importance of HCV treatment, regular health assessments, including monitoring of liver disease and specific lifestyle interventions to reduce liver disease-associated risk for this high-risk population. This approach may identify people with advancing chronic liver disease and improve their long-term health outcomes.

### Supplementary Information

**Supplementary Material 1.**

## Data Availability

The datasets used and/or analysed during the current study are available from the corresponding author upon reasonable request.

## Abbreviations

SUD: Substance use disorder
OUD: Opioid use disorder
OAT: Opioid agonist therapy
HCV: Hepatitis C virus
HIV: Human immunodeficiency virus
kPa: Kilopascal
cALCD: Compensated advanced chronic liver disease
BMI: Body mass index
iSUSI: Illegal Substance use severity index
RNA: Ribonucleic acid
HbA1c: Glycated haemoglobin
HDL: High-density lipoprotein
SD: Standard deviation
CI: Confidence interval
