# Supplementary material for: Liver stiffness and associated risk factors among people with a history of injecting drugs: a prospective cohort study
Source: Subst Abuse Treat Prev Policy. 2024 Mar 26;19:21. doi: 10.1186/s13011-024-00603-z (PMC10964694; doi:10.1186/s13011-024-00603-z)
Supplement: Supplementary file 1 — Supplementary Material 1. [file 13011_2024_603_MOESM1_ESM.docx]

**Supplementary Table 1: Baseline characteristics of the participants separated by OAT status (n=676)**

| \| *Sex n (%)* \| OAT (n=543) \| Non-OAT (n=133) \| Significance test† \| \| --- \| --- \| --- \| --- \| \| Females \| 154 (28) \| 31 (23) \| p= 0.24 \| \| Males \| 389 (72) \| 102 (77) \| \| *Age groups, years n (%)* \|  \|  \|  \| \| 18 - 29 \| 48 (8.8) \| 33 (25) \| p< 0.001 \| \| 30 - 39 \| 163 (30) \| 37 (28) \| \| 40 – 49 \| 171 (31) \| 29 (22) \| \| 50 - 59 \| 130 (24) \| 30 (23) \| \| 60 + \| 31 (5.7) \| 4 (3.0) \| \| Age, mean (SD) \| 44 (10) \| 40 (12) \| p< 0.001 \| \| BMI kg/m^2^, mean (SD) \| 25 (4.9) \| 24 (4.0) \| p=0.002 \| \| *Housing situation past 30 days^1^ n (%)* \|  \|  \|  \| \| Stable \| 490 (90) \| 97 (73) \| p< 0.001 \| \| Unstable \| 53 (10) \| 36 (27) \| \| *Highest completed education n* (%) \|  \|  \|  \| \| Not finished basic education^2^ \| 24 (4.4) \| 10 (7.5) \| p=0.099 \| \| Finished basic education^2^ \| 259 (48) \| 51 (38) \| \| High school^3^ \| 213 (39) \| 53 (40) \| \| < 3 years of higher education \| 37 (6.8) \| 14 (11) \| \| > 3 years of higher education \| 10 (1.8) \| 5 (3.8) \| \| *Current OAT medication n (%)* \|  \|  \|  \| \| Methadone \| 220 (33) \| - \|  \| \| Buprenorphine-based \| 322 (48) \| - \| \| Naltrexone \| 1 (0.2) \|  \| \| *Regular substance use^4^ n (%)* \|  \|  \|  \| \| Alcohol \| 121 (22) \| 42 (32) \| p=0.084 \| \| Tobacco \| 465 (86) \| 122 (92) \| p=0.62 \| \| Cannabis \| 247 (45) \| 77 (58) \| p=0.082 \| \| Stimulants \| 116 (21) \| 71 (53) \| p< 0.001 \| \| Opioids \| 55 (10) \| 44 (33) \| p< 0.001 \| \| Benzodiazepines \| 190 (35) \| 46 (35) \| p=0.47 \| \| iSUSI^5^, mean (SD) \| 0.33 (0.22) \| 0.46 (0.21) \|  \| \| Injected past 12 months^6^ n (%) \| 243 (45) \| 101 (76) \| p< 0.001 \| \| Hepatitis B antigen positive n (%) \| 3 (0,6) \| 1 (0.8) \|  \| \| HIV positive n (%) \| 3 (0,6) \| 0 \| \| *Hepatitis C status n (%)* \|  \|  \|  \| \| Antibody negative \| 25 (5) \| 31 (23) \| p< 0.001 \| \| Antibody positive, RNA negative \| 217 (40) \| 34 (26) \| \| RNA positive \| 270 (50) \| 40 (30) \| \| Missing information^7^ \| 31 (6) \| 28 (21) \| \| *Blood tests mean (SD)* \|  \|  \|  \| \| Alanine transaminase U/L \| 54 (68) \| 44 (59) \| p=0.17 \| \| Aspartate transaminase U/L \| 51 (51) \| 39 (30) \| p=0.024 \| \| Thrombocytes 10^9^/L \| 242 (81) \| 267 (67) \| p=0.004 \| \| Estimated glomerular filtration rate  ml/min/1,73m^2^ \| 107 (27) \| 105 (23) \| p=0.5 \| \| *Liver disease risk factors and markers* n (%) \|  \|  \|  \| \| HbA1c elevated^8^ \| 13 (2.4) \| 1 (0.8) \| p=0.32 \| \| Low HDL^9^ \| 166 (31) \| 7 (5.3) \| p< 0.001 \| \| Low thrombocytes^10^ \| 58 (11) \| 4 (3.0) \| p=0.02 \| \| Obesity^11^ ≥ 30 kg/m^2^ \| 96 (18) \| 8 (6.0) \| p= 0.001 \| \| Liver stiffness^12^ ≥ 10 kPa \| 78 (14) \| 5 (3.8) \| p= 0.001 \| |  |
| --- | --- | --- | --- | --- | --- | --- | --- | --- | --- | --- | --- | --- | --- | --- | --- | --- | --- | --- | --- | --- | --- | --- | --- | --- | --- | --- | --- | --- | --- | --- | --- | --- | --- | --- | --- | --- | --- | --- | --- | --- | --- | --- | --- | --- | --- | --- | --- | --- | --- | --- | --- | --- | --- | --- | --- | --- | --- | --- | --- | --- | --- | --- | --- | --- | --- | --- | --- | --- | --- | --- | --- | --- | --- | --- | --- | --- | --- | --- | --- | --- | --- | --- | --- | --- | --- | --- | --- | --- | --- | --- | --- | --- | --- | --- | --- | --- | --- | --- | --- | --- | --- | --- | --- | --- | --- | --- | --- | --- | --- | --- | --- | --- | --- | --- | --- | --- | --- | --- | --- | --- | --- | --- | --- | --- | --- | --- | --- | --- | --- | --- | --- | --- | --- | --- | --- | --- | --- | --- | --- | --- | --- | --- | --- | --- | --- | --- | --- | --- | --- | --- | --- | --- | --- | --- | --- | --- | --- | --- | --- | --- | --- | --- | --- | --- | --- | --- | --- | --- | --- | --- | --- | --- | --- | --- | --- | --- | --- | --- | --- | --- | --- | --- | --- | --- | --- | --- | --- | --- | --- |

† Chi square test for categorical variables and t-test for continuous variables

^1^ Living in an owned or rented home or being incarcerated was defined as a stable housing situation, while living in a homeless shelter, with family or friends, or on the street was defined as an unstable housing situation.

^2^ In Norway, the first ten school years are mandatory for all pupils.

^3^ Grades 11-13.

^4^ Substance used more regularly than once a week for the past 12 months.

^5^ The iSUSI is a continuous variable ranging from 0-1, were (1) indicates the effect of maximum substance use and (0) indicates the effect of no substance use.

^6^ Intravenous injection of drugs at least once during the past 12 months.

^7^ No information on hepatitis C status registered.

^8^ Defined as HbA1c > 48 mmol/mol, which is diagnostic of diabetes (1).

^19^ Values below 1.3 mmol/L for women and 1.0 mmol/L for men are defined as low (2, 3).

^10^ Thrombocyte count of < 150 x 10^9^/L is defined as low and is associated with an increased risk of portal hypertension (4)

^11^ BMI ≥ 30 kg/m^2^ increases the risk of severe liver disease outcome (5)

^12^Liver stiffness ≥ 10 kPa indicates an increased risk of compensated advanced chronic liver disease (4).

**Supplementary table 2: Overview of missing data per participant ( n = 676) and variable (n = 11)**

| **Per participant** | |  | **Per variable** | |
| --- | --- | --- | --- | --- |
| No. of missing values | n (%) |  | Variable | n (%) |
| 0 | 270 (40%) |  | Hepatitis C RNA | 115 (17) |
| 1 | 291 (43%) |  | Low HDL Cholesterol | 95 (14) |
| 2 | 22 (3%) |  | HbA1c | 60 (8.9) |
| 3 | 50 (7%) |  | Injected last 12 months | 51 (7.5) |
| 4 | 0 |  | Alcohol last 12 months | 44 (6.5) |
| 5 | 18 (3%) |  | Benzodiazepines last 12 months | 44 (6.5) |
| 6 | 16 (2%) |  | Cannabis last 12 months | 44 (6.5) |
| 7 | 0 |  | Opioids last 12 months | 44 (6.5) |
| 8 | 10 (1%) |  | Stimulants last 12 months | 44 (6.5) |
|  |  |  | Tobacco last 12 months | 44 (6.5) |
|  |  |  | BMI | 10 (1.5) |

**Supplementary table 3: Linear mixed model of risk factors predicting liver stiffness ≥ 10 (kPa) (n=676)**

| **Fixed effects** |  |  |
| --- | --- | --- |
|  | **Effect estimate** | **Time trend (per year)** |
|  | Coefficient (95% CI) | Coefficient (95% CI) |
| Yearly LSM change | - | **0.28 (0.083; 0.47)*** |
| Age per 10 years increase | **0.070 (0.049; 0.092)*** | -0.013 (-0.037; 0.010) |
| Regular alcohol use^1^ | **0.076 (0.024; 0.13)*** | -0.0014 (-0.056; 0.053) |
| High substance use^2^ | 0.038 (-0.070; 0.15) | -0.032 (-0.15; 0.092) |
| Body mass index (kg/m^2^) | **0.015 (0.010; 0.020)*** | **-0.0084 (-0.014; -0.0026)*** |
| Hepatitis C RNA positive^3^ | **0.086 (0.041; 0.13)*** | - |
| Low HDL Cholesterol^4^ | **0.085 (0.032; 0.14)*** | -0.0086 (-0.064; 0.047) |
| Elevated HbA1c^5^ | 0.15 (-0.0059; 0.31) | 0.16 (-0.37; 0.36) |
| Hepatitis C status change^6^ | - | **-0.053 (-0.098; -0.0071)*** |

The table shows a linear mixed model analysis of how the exposure variables predicts a dichotomous liver stiffness outcome defined as < 10 kPa (0) and ≥ 10 kPa (1). The constant term (β_0_) was -0.67 (-0.84; -0.50). Except for HCV status, the time trend indicates the effect of the variable remaining at baseline levels over time. Except for HCV status, the time trend indicates the effect of the variable remaining at baseline levels over time. Significantly associated (p< 0.05) variables are labelled with bold text and an asterisk.

^1^Using alcohol on one or more days per week per year

^2^The iSUSI is a continuous variable ranging from 0-1, were (1) indicates the effect of maximum substance use and (0) indicates the effect of no substance use.

^3^Hepatitis C virus RNA positive at baseline

^4^Below 1.3 mmol/L for women and 1.0 mmol/L for men

^5^Above 48 mmol/mol, indicating diabetes mellitus

^6^Resolved hepatitis C infection compared with no change in HCV infection status from baseline to the following liver stiffness measurements

**Supplementary table 4: Adjusted linear mixed model of liver stiffness (kPa) for all persons in sample and only persons with BMI < 30 kg/m^2^ BMI < 30kg/m^2^.**

|  | Only persons with BMI < 30 kg/m^2^ | | All persons | |
| --- | --- | --- | --- | --- |
| **Fixed effects** | **Effect estimate** | **Time trend** | **Effect estimate** | **Time trend** |
|  | Estimate (95% CI) | Estimate (95% CI) | Estimate (95% CI) | Estimate (95% CI) |
| Yearly LSM change | - | 0.91 (-1.63; 3.4) | - | **2.81 (0.56; 5.0)*** |
| Age per 10 years increase | **0.92 (0.61; 1.2)*** | -0.11 (-0.37; 0.16) | **1.0 (0.68; 1.3)*** | -0.18 (-0.45; 0.088) |
| Regular alcohol use^1^ | 0.75 (-0.019; 1.5) | 0.11 (-0.51; 0.72) | **1.3 (0.46; 2.0)*** | -0.046 (-0.67; 0.58) |
| High substance use^2^ | 0.45 (-1.1; 2.0) | -0.56 (-1.97; 0.84) | 0.66 (-0.96; 2.3) | -0.48 (-1.9; 0.94) |
| Body mass index (kg/m^2^) | **0.16 (0.054; 0.26)*** | -0.013 (-0.097; 0.070) | **0.25 (0.17; 0.32)*** | **-0.079 (-0.14; -0.013)*** |
| Hepatitis C RNA positive^3^ | **1.2 (0.50; 1.9)*** | - | **1.2 (0.54; 1.9)*** | - |
| Low HDL Cholesterol^4^ | **0.94 (0.14; 1.7)*** | -0.45 (-1.1; 0.19) | **1.4 (0.64; 2.2)*** | -0.55 (-1.2; 0.08) |
| Elevated HbA1c^5^ | 2.6 (-0.24; 5.5) | **14 (8.7; 19)*** | **3.1 (0.68; 5.5)*** | **4.6 (2.3; 6.9)*** |
| Hepatitis C status change^6^ | - | **-0.62 (-1.1; -0.11)*** | - | **-0.73 (-1.3; -0.21)*** |

The constant term (β_0_) was -5.4 (CI -8.0; -2.8) for all persons and -2.6 (-5.6; 0.39) for persons with BMI < 30kg/m2.

Except for HCV status, the time trend indicates the effect of the variable remaining at baseline levels over time. Significantly associated (p< 0.05) variables are labelled with bold text and an asterisk.

^1^Using alcohol on one or more days per week per year

^2^The iSUSI is a continuous variable ranging from 0-1, were (1) indicates the effect of maximum substance use and (0) indicates the effect of no substance use.

^3^Hepatitis C virus RNA positive at baseline

^4^Below 1.3 mmol/L for women and 1.0 mmol/L for men

^5^Above 48 mmol/mol, indicating diabetes mellitus

^6^Resolved hepatitis C infection compared with no change in HCV infection status from baseline to the following liver stiffness measurements

**Supplementary Table 5: Adjusted linear mixed model of liver stiffness (kPa) for all persons in sample with sex as a predictor variable (n = 676).**

|  |  | |
| --- | --- | --- |
| **Fixed effects** | **Effect estimate** | **Time trend** |
|  | Estimate (95% CI) | Estimate (95% CI) |
| Yearly LSM change | - | **2.7 (0.43; 5.0)*** |
| Age per 10 years increase | **0.99 (0.66; 1.3)*** | -0.17 (-0.44; 0.095) |
| Not female sex | -0.51 (-1.3; 0.27) | 0.12 (-0.54; 0.79) |
| Regular alcohol use^1^ | **1.2 (0.44; 2.0)*** | -0.047 (-0.67; 0.58) |
| High substance use^2^ | 0.58 (-1.1; 2.2) | -0.44(-1.9; 0.98) |
| Body mass index (kg/m^2^) | **0.24 (0.17; 0.32)*** | **-0.077 (-0.14; -0.011)*** |
| Hepatitis C RNA positive^3^ | **1.2 (0.50; 1.9)*** | - |
| Low HDL Cholesterol^4^ | **1.6 (0.74; 2.4)*** | -0.59 (-1.2; 0.065) |
| Elevated HbA1c^5^ | **3.1 (0.68; 5.5)*** | **4.6 (2.3; 6.9)*** |
| Hepatitis C status change^6^ | - | **-0.73 (-1.3; -0.20)*** |

The constant term (β_0_) was -5.0 (-7.6; -2.3). Except for HCV status, the time trend indicates the effect of the variable remaining at baseline levels over time. Significantly associated (p< 0.05) variables are labelled with bold text and an asterisk.

^1^Using alcohol on one or more days per week per year

^2^The iSUSI is a continuous variable ranging from 0-1, were (1) indicates the effect of maximum substance use and (0) indicates the effect of no substance use.

^3^Hepatitis C virus RNA positive at baseline

^4^Below 1.3 mmol/L for women and 1.0 mmol/L for men

^5^Above 48 mmol/mol, indicating diabetes mellitus

^6^Resolved hepatitis C infection compared with no change in HCV infection status from baseline to the following liver stiffness measurements

**Supplementary Table 6 : Adjusted linear mixed model of liver stiffness (kPa) without missing values (n = 500).**

|  |  | |
| --- | --- | --- |
| **Fixed effects** | **Effect estimate** | **Time trend** |
|  | Estimate (95% CI) | Estimate (95% CI) |
| Yearly LSM change | - | **3.1 (0.85; 5.4)*** |
| Age per 10 years increase | **1.1 (0.72; 1.5)*** | -0.20 (-0.47; 0.077) |
| Regular alcohol use^1^ | **1.7 (0.80; 2.6)*** | -0.12 (-0.76; 0.52) |
| High substance use^2^ | 0.81 (-0.97; 2.6) | -0.72 (-2.2; 0.71) |
| Body mass index (kg/m^2^) | **0.26 (0.18; 0.35)*** | **-0.081 (-0.15; -0.014)*** |
| Hepatitis C RNA positive^3^ | **1.5 (0.78; 2.3)*** | - |
| Low HDL Cholesterol^4^ | **1.5 (0.61; 2.3)*** | **-0.67 (-1.3; -0.020)*** |
| Elevated HbA1c^5^ | **4.8 (2.0; 7.5)*** | 2.2 (-0.27; 4.8) |
| Hepatitis C status change^6^ | - | **-0.82 (-1.4; -0.29)*** |

The constant term (β_0_) was -6.4 (-9.3; -3.5).

Except for HCV status, the time trend indicates the effect of the variable remaining at baseline levels over time. Significantly associated (p< 0.05) variables are labelled with bold text and an asterisk.

^1^Using alcohol on one or more days per week per year

^2^The iSUSI is a continuous variable ranging from 0-1, were (1) indicates the effect of maximum substance use and (0) indicates the effect of no substance use.

^3^Hepatitis C virus RNA positive at baseline

^4^Below 1.3 mmol/L for women and 1.0 mmol/L for men

^5^Above 48 mmol/mol, indicating diabetes mellitus

^6^Resolved hepatitis C infection compared with no change in HCV infection status from baseline to the following liver stiffness measurements

**Supplementary Table 7: Adjusted linear mixed model of liver stiffness (kPa) for all persons in sample with sex as a predictor variable, without missing** **values (n = 500).**

|  |  | |
| --- | --- | --- |
| **Fixed effects** | **Effect estimate** | **Time trend** |
|  | Estimate (95% CI) | Estimate (95% CI) |
| Yearly LSM change | - | **3.1 (0.75; 5.4)*** |
| Age per 10 years increase | **1.1 (0.71; 1.5)*** | -0.20 (-0.48; 0.080) |
| Not female sex | **-0.88 (-1.7; -0.019)*** | 0.095 (-0.58; 0.77) |
| Regular alcohol use^1^ | **1.7 (0.78; 2.6)*** | -0.12 (-0.76; 0.52) |
| High substance use^2^ | 0.67 (-1.1; 2.4) | -0.70(-2.1; 0.75) |
| Body mass index (kg/m^2^) | **0.26 (0.17; 0.34)*** | **-0.080 (-0.15; -0.012)*** |
| Hepatitis C RNA positive^3^ | **1.5 (0.72; 2.2)*** | - |
| Low HDL Cholesterol^4^ | **1.6 (0.76; 2.5)*** | **-0.69 (-1.4; -0.030)*** |
| Elevated HbA1c^5^ | **4.8 (2.0; 7.5)*** | 2.3 (-0.25; 4.8) |
| Hepatitis C status change^6^ | - | **-0.81 (-1.4; -0.27)*** |

The constant term (β_0_) was -5.9 (-8.8; -2.9). Except for HCV status, the time trend indicates the effect of the variable remaining at baseline levels over time. Significantly associated (p< 0.05) variables are labelled with bold text and an asterisk.

^1^Using alcohol on one or more days per week per year

^2^The iSUSI is a continuous variable ranging from 0-1, were (1) indicates the effect of maximum substance use and (0) indicates the effect of no substance use.

^3^Hepatitis C virus RNA positive at baseline

^4^Below 1.3 mmol/L for women and 1.0 mmol/L for men

^5^Above 48 mmol/mol, indicating diabetes mellitus

^6^Resolved hepatitis C infection compared with no change in HCV infection status from baseline to the following liver stiffness measurements

**Supplementary table 8: Adding regular tobacco use as independent variable (n = 676).**

|  |  | |
| --- | --- | --- |
| **Fixed effects** | **Effect estimate** | **Time trend** |
|  | Estimate (95% CI) | Estimate (95% CI) |
| Yearly LSM change | - | **2.8 (0.18; 5.4)*** |
| Age per 10 years increase | **0.98 (0.65; 1.3)*** | -0.18 (-0.45; 0.091) |
| Regular alcohol use^1^ | **1.2 (0.45; 2.0)*** | -0.044 (-0.067; 0.58) |
| Regular tobacco use^1^ | -1.1 (-2.5; 0.23) | 0.032 (-1.1; 1.2) |
| High substance use^2^ | 0.72 (-0.91; 2.3) | -0.48 (-1.9; 0.94) |
| Body mass index (kg/m^2^) | **0.23 (0.17; 0.32)*** | **-0.079 (-0.14; -0.012)*** |
| Hepatitis C RNA positive^3^ | **1.2 (0.556;1.9)*** | - |
| Low HDL Cholesterol^4^ | **1.5 (0.68; 2.3)*** | -0.55 (-1.2; 0.087) |
| Elevated HbA1c^5^ | **3.1 (0.72; 5.5)*** | **4.6 (2.3; 6.9)*** |
| Hepatitis C status change^6^ | - | **-0.73 (-1.2; -0.20)*** |

Except for HCV status, the time trend indicates the effect of the variable remaining at baseline levels over time. Significantly associated (p< 0.05) variables are labelled with bold text and an asterisk.

^1^Using alcohol or tobacco on one or more days per week per year

^2^The iSUSI is a continuous variable ranging from 0-1, were (1) indicates the effect of maximum substance use and (0) indicates the effect of no substance use.

^3^Hepatitis C virus RNA positive at baseline

^4^Below 1.3 mmol/L for women and 1.0 mmol/L for men

^5^Above 48 mmol/mol, indicating diabetes mellitus

^6^Resolved hepatitis C infection compared with no change in HCV infection status from baseline to the following liver stiffness measurements

References:

1. Organization WH. Diagnosis and management of type 2 diabetes (HEARTS-D). Geneva; 2020.

2. EASL-EASD-EASO Clinical Practice Guidelines for the management of non-alcoholic fatty liver disease. J Hepatol. 2016;64(6):1388-402.

3. Chalasani N, Younossi Z, Lavine JE, Charlton M, Cusi K, Rinella M, et al. The diagnosis and management of nonalcoholic fatty liver disease: Practice guidance from the American Association for the Study of Liver Diseases. Hepatology. 2018;67(1):328-57.

4. de Franchis R, Bosch J, Garcia-Tsao G, Reiberger T, Ripoll C. Baveno VII - Renewing consensus in portal hypertension. J Hepatol. 2022;76(4):959-74.

5. Jarvis H, Craig D, Barker R, Spiers G, Stow D, Anstee QM, Hanratty B. Metabolic risk factors and incident advanced liver disease in non-alcoholic fatty liver disease (NAFLD): A systematic review and meta-analysis of population-based observational studies. PLoS Med. 2020;17(4):e1003100.
